# Experiences with home monitoring technology in older adults with traumatic brain injury: a qualitative study

**DOI:** 10.1186/s12877-024-05397-0

**Published:** 2024-09-30

**Authors:** Megan E. Parkinson, Rebecca M. Smith, Karen Tanious, Francesca Curtis, Rebecca Doherty, Lorena Colon, Lucero Chena, Sophie C. Horrocks, Matthew Harrison, Michael B. Fertleman, Melanie Dani, Payam Barnaghi, David J. Sharp, the UK Dementia Research Institute Care Research & Technology Research Group, Lucia M. Li

**Affiliations:** 1 Dementia Research Institute Care Research and Technology Centre, Imperial College London and the University of Surrey, London, UK; 2 Department of Bioengineering, Imperial College London, London, UK; 3 Department of Brain Sciences, Imperial College London, London, UK

**Keywords:** Remote monitoring, Acceptability, Technology, Traumatic brain injury

## Abstract

**Background:**

Home monitoring systems utilising artificial intelligence hold promise for digitally enhanced healthcare in older adults. Their real-world use will depend on acceptability to the end user i.e. older adults and caregivers. We explored the experiences of adults over the age of 60 and their social and care networks with a home monitoring system installed on hospital discharge after sustaining a moderate/severe Traumatic Brain Injury (TBI), a growing public health concern.

**Methods:**

A qualitative descriptive approach was taken to explore experiential data from older adults and their caregivers as part of a feasibility study. Semi-structured interviews were conducted with 6 patients and 6 caregivers (*N* = 12) at 6-month study exit. Data were analysed using Framework analysis. Potential factors affecting acceptability and barriers and facilitators to the use of home monitoring in clinical care and research were examined.

**Results:**

Home monitoring was acceptable to older adults with TBI and their caregivers. Facilitators to the use of home monitoring were perceived need for greater support after hospital discharge, the absence of sound and video recording, and the peace of mind provided to care providers. Potential barriers to adoption were reliability, lack of confidence in technology and uncertainty at how data would be acted upon to improve safety at home.

**Conclusions:**

Remote monitoring approaches are likely to be acceptable, especially if patients and caregivers see direct benefit to their care. We identified key barriers and facilitators to the use of home monitoring in older adults who had sustained TBI, which can inform the development of home monitoring for research and clinical use. For sustained use in this demographic the technology should be developed in conjunction with older adults and their social and care networks.

**Supplementary Information:**

The online version contains supplementary material available at 10.1186/s12877-024-05397-0.

## Introduction

### Passive home monitoring

Passive home monitoring systems incorporating sensors and artificial intelligence have shown promise in deriving indicators of health and function by analysing changes in patients' activity and sleep patterns [1–3]. Home sensors can collect vast amounts of data over long periods, potentially enabling early detection of changes in older adults’ health before crisis point [1, 2, 4]. The ability to generate markers of health and function from these systems could facilitate targeted interventions from community health and social care teams. Thus, these systems offer an attractive solution for digitally enhanced healthcare, promoting independence among older adults, and reducing hospital admissions and institutionalisation [1, 4–7]. They may be particularly useful for vulnerable populations where insight is impaired [1, 4–7]. For sustainable adoption of home monitoring in care and research, it is vital to understand the experiences of older adults and their caregivers with these technologies [8].

### Traumatic Brain Injury (TBI)

Traumatic Brain Injury (TBI) presents a significant global health challenge. It affects ~ 55 million people worldwide and is a leading cause of trauma related death and disability, with annual global health care costs estimated at £350 billion [9]. Historically, TBI has been associated with younger patients and high-energy traumas, such as road traffic accidents. However, an epidemiological shift has been observed in high-income countries, with TBI incidence among older adults increasing faster than any other age group, primarily due to falls [10–21].The period immediately following hospital discharge is particularly critical for older adults, who may be especially vulnerable to complications. Notably, 40% of hospital readmissions for older adults with TBI occur within 30 days of the initial trauma [22]. Despite this, older adults are underrepresented in TBI research, and specialist follow up and support post-discharge are limited [23, 24].

### Qualitative studies on home monitoring

Qualitative studies examining patient and caregiver experiences with home monitoring systems have primarily focused on single occupancy households with independently living older adults [25–30]. There remains a gap in research concerning adults living with significant frailty, cognitive impairment or those living in multi occupancy settings. In addition, only one study has looked at the experience of older adults with home monitoring during the transition from hospital inpatient to their own homes [31]. Understanding the experiences of older adults in these more realistic settings is essential. Our study extends the literature by focusing on perceptions of the equipment used during the period from hospital to home after a TBI and explores the experiences of older adults and caregivers, including those living with frailty, cognitive impairment and in multi-occupancy households.

### Study Aims

Our qualitative study aims to explore the experiences of older adult TBI patients and caregivers with home monitoring technology, with a focus on:

- Acceptability and tolerability.
- Barriers to and facilitators of the use of these systems clinically and in research.

## Methods

### The home monitoring intervention

**Fig. 1:**
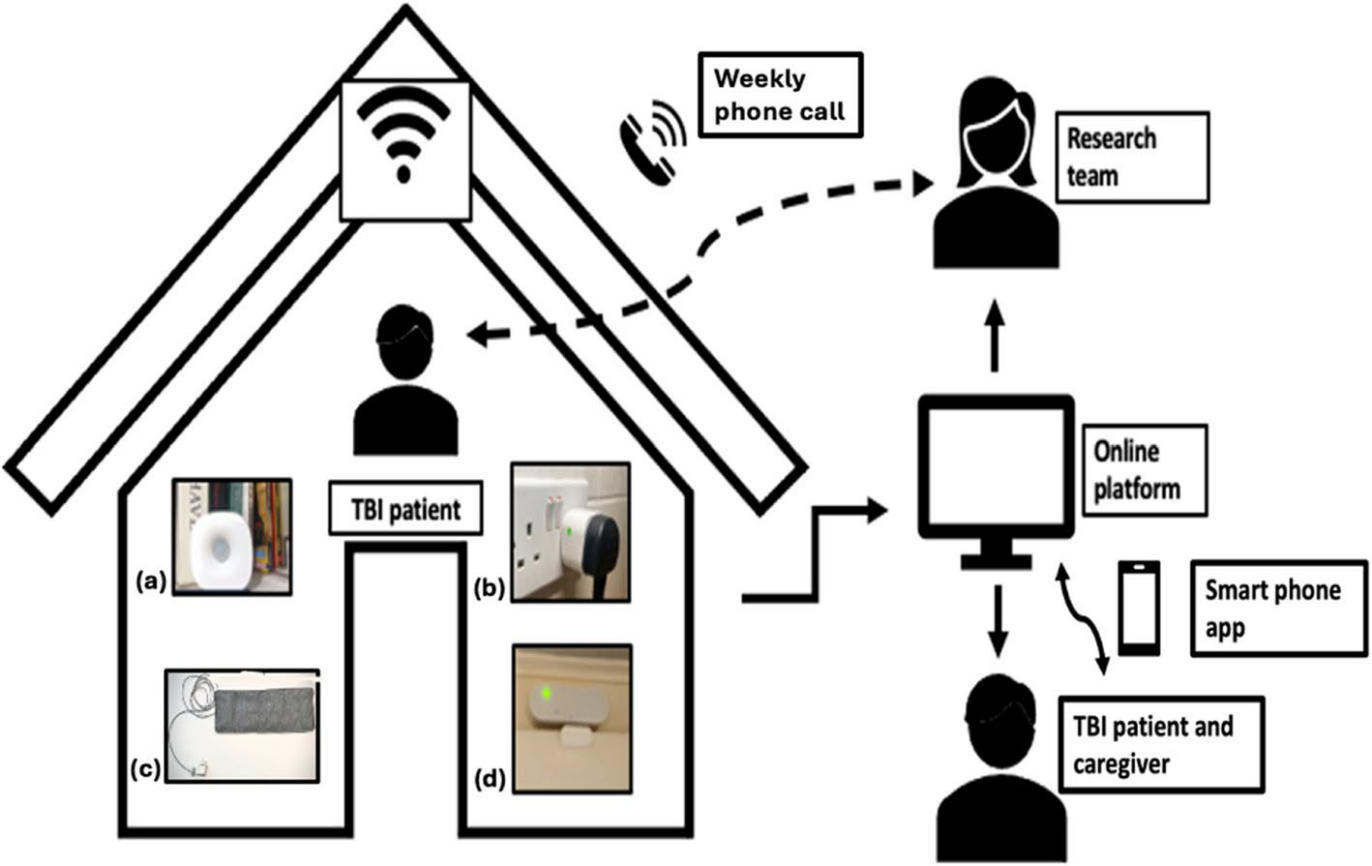
The Home Monitoring Set up. Passive infrared sensors (PIR) (**a**) measure light, temperature and heat [33],they sense movement up to 9 m away from the sensor [33]. Smart plugs (**b**) record electrical use as a proxy for appliance use. The Withing’s bed mat (**C**) passively captures minute-by-minute heart rate, respiratory rate and movement using pneumatic sensors. The bed mat is waterproof and is placed out of sight underneath the mattress. Door sensors (**d**) record any opening or closing. A base unit receives binary data from the sensors via a wireless protocol and sends it to the cloud in real time. In our feasibility study, the data was not viewed live by the research team

This qualitative study formed part of a feasibility study evaluating the use of a home monitoring system to study the effects of TBI in older adults, after hospital discharge. The system included a bed mat [32], door sensors [33], smart plugs, and passive infrared (PIR) movement sensors [33], placed in the most active areas of the home. The set-up is summarised in Fig. 1 and further detail is found in the study protocol [34]. Inclusion and exclusion criteria for the study are listed in Table 1.

**Table 1:** Inclusion and exclusion criteria for the feasibility study

Inclusion	Exclusion
• Aged over 60 • Moderate/severe TBI based on the MAYO criteria [35] • Equipment can be installed 3 weeks from hospital discharge	**• **Discharge to location other than own home **• **Unstable medical condition or severe disability significantly affecting recovery **• **Remote repatriation or home address

Sensors collected movement and bed mat data over six months, providing aggregate measures to track household activity and sleep. Additionally, patients were offered a wearable activity watch for personal activity tracking. Caregivers could view the sensor data via a smartphone application. In our feasibility study, there was no live monitoring and feedback, this was made explicitly clear to patients and study contacts during the consenting process. Our home monitoring system is a simplified version of the Minder program, a monitoring system used by the UK Dementia Research Institute Care Research and Technology Centre (DRI CR&T) for home monitoring in dementia patients, which contains live monitoring and feedback during the weekday daytime [36].

### Research team

Data collection was undertaken by three female clinical academics. MP is a Registrar in Geriatric Medicine and a Clinical Research Fellow. LML is a Registrar in Neurology and post-doctoral Clinical Lecturer, with a specialist interest in TBI. FC holds a master’s in clinical psychology. RS a Clinical Academic Physiotherapist and NIHR Imperial BRC Post-doctoral Research Fellow, with previous experience in qualitative research provided oversight. Researchers conducting interviews did not have qualitative training however had experience conducting interviews in previous work.

### Patient and public involvement

People living with dementia (PLWD) and carers were involved in the design of the wider Minder program and study, through the "Minder Champion" program. Minder Champions provided feedback on devices, sensors and participant experiences. They tested new devices with additional support and contributed feedback through focus groups before broader implementation.

### Study design

The study employed a qualitative descriptive methodology. This approach is suited to the complexity and variability of studies in healthcare and is advantageous for its ability to deliver a comprehensive account of participants experiences in their own words [37]. It is also well-suited for topics with limited research, providing a foundation for future studies [37, 38]. Semi-structured interviews were conducted using an interview guide, which were then analysed using framework analysis.

The study was granted ethical approval by the London—Camberwell St Giles Research Ethics Committee (REC number: 17/LO/2066). Our study was conducted in accordance with the Declaration of Helsinki.

### Participant selection

Convenience sampling was used, including all willing study participants for interviews. To be eligible, patients had to have consented to the wider feasibility study (*n* = 12 households) and used the home monitoring system for the full 6-month period. Written consent was obtained in person. If patients lacked capacity, they could be enrolled with permission from a next of kin or nominated consultee. Patients' nominated study contacts, such as family and carers, were also invited to interviews. The sample size was informed by previous studies [39–42]. Nineteen people were invited to interviews—9 patients and 10 study contacts. A total of 12 were recruited and interviewed—6 patients and 6 study contacts. The 7 who did not participate included 1 patient who declined, 2 patients who had poor cognition and 4 study contacts who could not commit the time required to interview.

### Setting

To facilitate participation, interviews were offered face-to-face or remotely by phone. Patients and study contacts were interviewed together or separately based on their preference. Interviews were carried out in patients’ homes or in TBI clinic.

### Data collection

Data were collected through semi-structured interviews conducted at the end of the patients monitoring period in the feasibility study. The interview guide was informed by previous qualitative studies on older adults’ experiences with home monitoring equipment [30, 43–45] and developed using the Theoretical Domains Framework (TDF), a tool designed to understand factors influencing behaviour by examining aspects like beliefs, knowledge and social influences [46]. The use of this framework ensured a thorough exploration of the factors that may influence acceptability and the barriers and facilitators to use of the home monitoring system [47]. Separate interview guides were developed for patients and study contacts. The full interview guides are included in Supplementary file 1. Interviews were audio recorded and transcribed verbatim using BUZZ (transcription software) [48]. For practical reasons, and because the qualitative study was exploratory, the interviews were not pretested, and participant responses were not validated with them after interview.

### Data analysis

Framework analysis was chosen for its structured approach to data organisation, ensuring rigorous analysis. This method also easily facilitates comparisons across different themes, making it effective in identifying barriers and facilitators [49, 50]. Step by step analysis was carried out until a clear account of the data emerged. Guidance from key qualitative resources was used [49, 51]. Participants did not provide feedback on the findings from our analysis. The steps of framework analysis are outlined below.

### Familiarisation

MP, FC, LL, LC and LC familiarised themselves with the interview material by listening to and re-reading the transcripts and making notes.

### Identifying a thematic framework

MP, FC, LL, and LC each reviewed the transcripts individually, then met as a group to discuss and identify key themes in the data. These themes were assigned single-word codes and grouped into similar categories to form a thematic framework. A deductive approach, guided by the TDF, was used, while also allowing inductive themes to emerge from the data to ensure the findings were not limited to the TDF's predefined categories [52]. Data saturation was sought and agreed upon when no new codes were added from the last 3 transcripts. MP reviewed three iterations of this framework to ensure it comprehensively covered the entire dataset. The final thematic framework is included in Supplementary file 2.

### Indexing

MP systematically applied the framework to the transcripts using NVivo software (V.14). Each piece of interview data was indexed with one or more themes that best represented its content, helping to identify consistencies in the data. For example, all mentions of privacy concerns were tagged with the theme “privacy concerns”.

### Charting

**Fig. 2:**
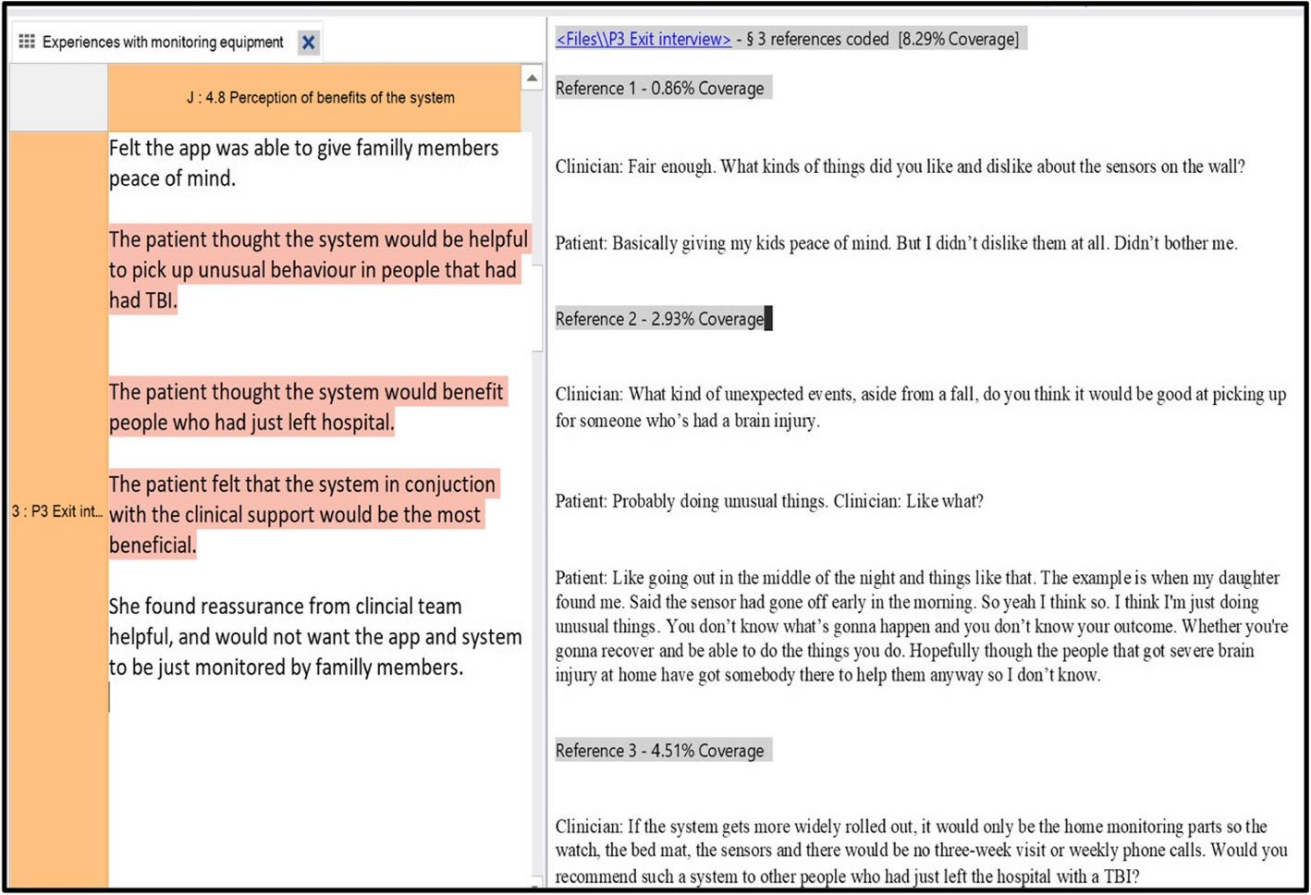
An extract from NVIVO showing the framework and transcript. The columns show the categories from the framework and the rows summaries of interviewees responses with example quotes This creates a chart where the interviewee’s responses are summarised and sorted according to the framework categories. This allows summaries to be read downwards for analysis of a certain theme or between cases

Using NVivo (V.14) a matrix was created with participants as rows and themes as columns. Relevant data were summarised or quoted in the cells of the matrix, representing how each participant's experiences related to each theme. Figure 2 shows an example of the charting process.

### Mapping and interpretation

The matrix was examined to identify patterns and relationships between themes. Common themes were described and then categorised into potential barriers and facilitators for adopting and this technology as well as factors affecting acceptability. We evaluated acceptability using the Unified Theory of Acceptance and Use of Technology (UTAUT), which considers factors such as effort expectancy, perceived usefulness and social support [53].

## Results

This study has been reported in accordance with the Consolidated Criteria for reporting Qualitative Research (COREQ) [54] checklist (Supplementary file 3).

### Participants

Interviews lasted between 30 and 90 min. Our study group included older adults with variations in frailty, multimorbidity, functional ability, cognitive function, educational attainment, and socio-economic status. Patients' demographics are included in Table 2. Patients in the study were aged between 67 and 96, with a median age of 83. Five (55%) patients were living with very mild and mild frailty, whilst 2 (22%) were living with very severe frailty, defined by the Clinical Frailty Score (CFS). The average Charlson Comorbidity Index (CCI) was 8 (85% 1-year mortality). The average Barthel Index was 83 (scores of 61–90 indicate moderate dependency). Two (22%) had a Montreal Cognitive Assessment score (MOCA) indicative of mild cognitive impairment, and 2 (22%) had a formal diagnosis of dementia. Five (55%) had university education or higher. Six (66%) patients lived in multi-occupancy residences, and 3 (44%) lived alone. Four (40%) of the study contacts lived with study patients. The study contact’s relationship to the patient is included in Table 3.

**Table 2:** Participant demographics and reason for not participating

Household number	Age (Sex)	CFS	CCI	Barthel	MOCA	Education level	Socio-economic class	Participated in interview (reason if declined)	Receiving home care	Type of household	Technology
P1	86 (M)	5	9	100	30	University	Higher managerial, administrative, and professional occupations	Yes	No	Multiple occupancy	Passive sensors
P2	96 (M)	7	8	50	15	University	Higher managerial, administrative, and professional occupations	No (Poor cognition. Ill health)	Yes	Multiple occupancy	Passive sensors
P3	67 (F)	2	4	100	29	School leaver	Routine and manual occupations	Yes	No	Single occupancy	Passive sensors And wearable
P4	79 (M)	4	4	100	24	University	Higher managerial, administrative, and professional occupations	Yes	Yes	Multiple occupancy	Passive sensors
P5	84 (F)	5	5	95	23	School leaver	Routine and manual occupations	Yes	Yes	Multiple occupancy	Passive sensors
P6	70 (F)	1	3	100	25	School leaver	Intermediate occupations	Yes	No	Multiple occupancy	Passive sensors and wearable
P7	64 (F)	7	3	5	0	University	Higher managerial, administrative, and professional occupations	No (Poor cognition. Ill health)	Yes	Multiple occupancy	Passive sensors
P8	83 (M)	4	4	100	22	School leaver	Routine and manual occupations	No (Declined to be recorded)	Declined homecare	Single occupancy	Passive sensors and wearable
P9	87 (M)	4	8	100	25	University	Higher managerial, administrative, and professional occupations	Yes	No	Single occupancy	Passive sensors

Rockwood Clinical Frailty Scale (CFS) [55], Comorbidity Index (CCI), Montreal cognitive assessment (MOCA) [56], Barthel Index of Activities of Daily Living (Barthel) [57], Socio-economic class refers to the National Statistics Socio-economic classification (NS-SEC)- Office for National Statistics [58]

**Table 3:** Study contact demographics and reason for not participating

Study contact	Age	Sex	Relationship to participant	Cohabited with participant	Participated in interview	Reason for study contact not participating
P1 study contact	86	F	Wife	Yes	Yes	-
P2 study contact	89	F	Wife	Yes	Yes	-
	NR	F	Daughter	Yes	No	Other commitments
P3 study contact	NR	F	Daughter	No	No	Other commitments
P4 study contact	79	F	Wife	Yes	Yes	-
P5 study contact	54	F	Daughter	No	Yes	-
P6 study contact	NR	M	Husband	Yes	No	Other commitments
P7 study contact	63	M	Husband	Yes	yes	
P8 study contact	87	F	Friend	No	Yes	-
P9 study contact	NR	M	Son	No	No	Other commitments

*NR* Not recorded

### Overview of themes

**Fig. 3:**
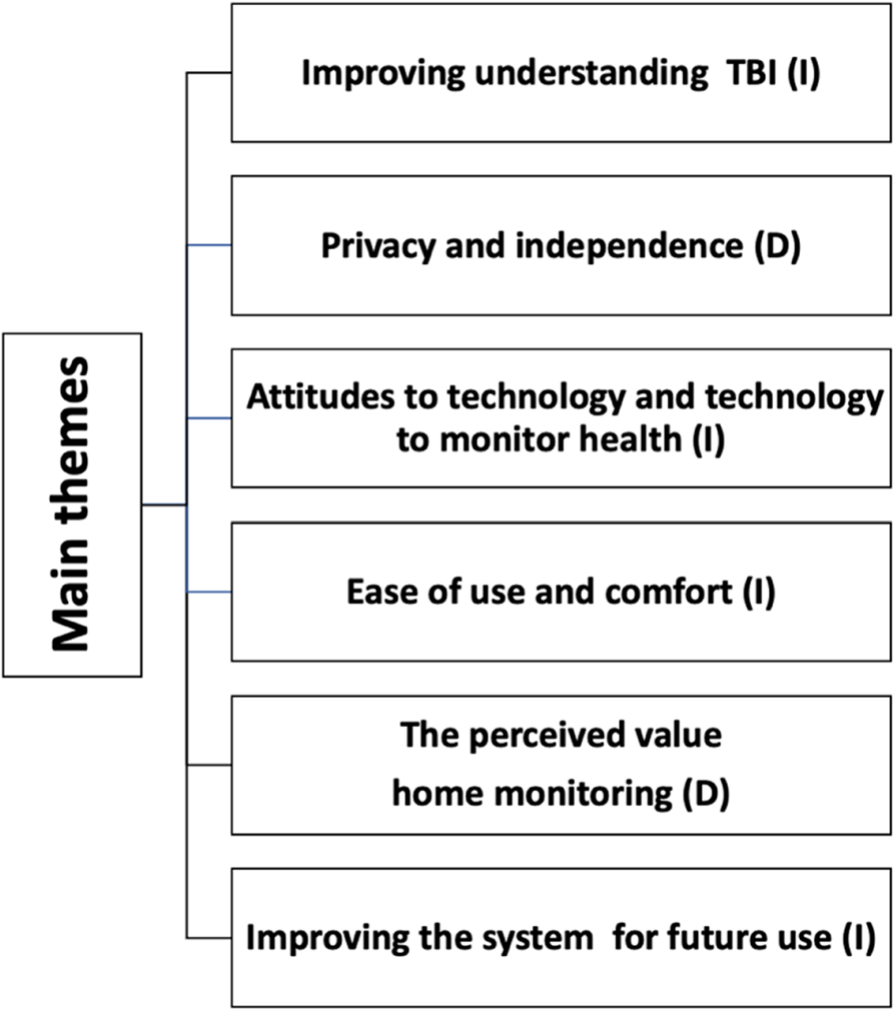
Main themes from the interview material. Diagram highlighting the 6 main themes that emerged from the interviews relating to experiences with the monitoring equipment. (I) highlights themes that were produced inductively and (D) to highlight deductive themes

**Fig. 4:**
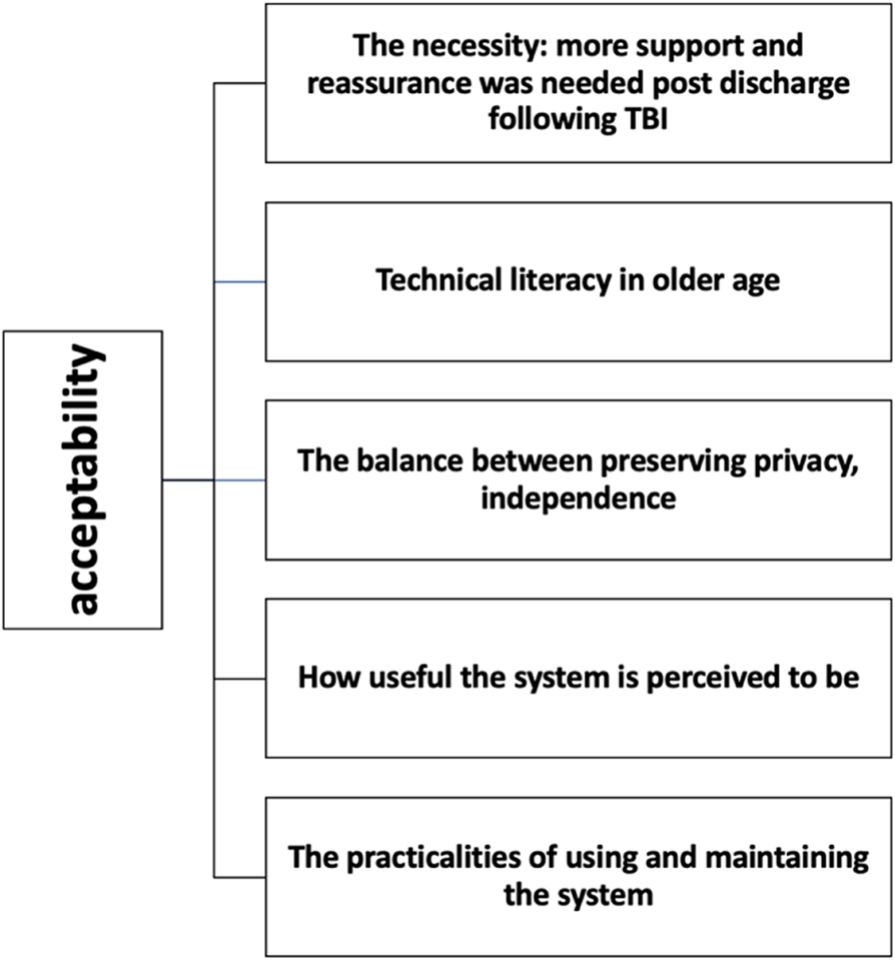
Factors we found affecting the acceptability of home monitoring. Factors we found affecting the acceptability of home monitoring are outlined. These themes align with the Unified Theory of Acceptance of Technology use (UTAUT) [53], which proposes that acceptance of technology is affected by effort expectancy, how useful it is perceived to be and the degree to which the technology is supported by the individual’s social network

**Fig. 5:**
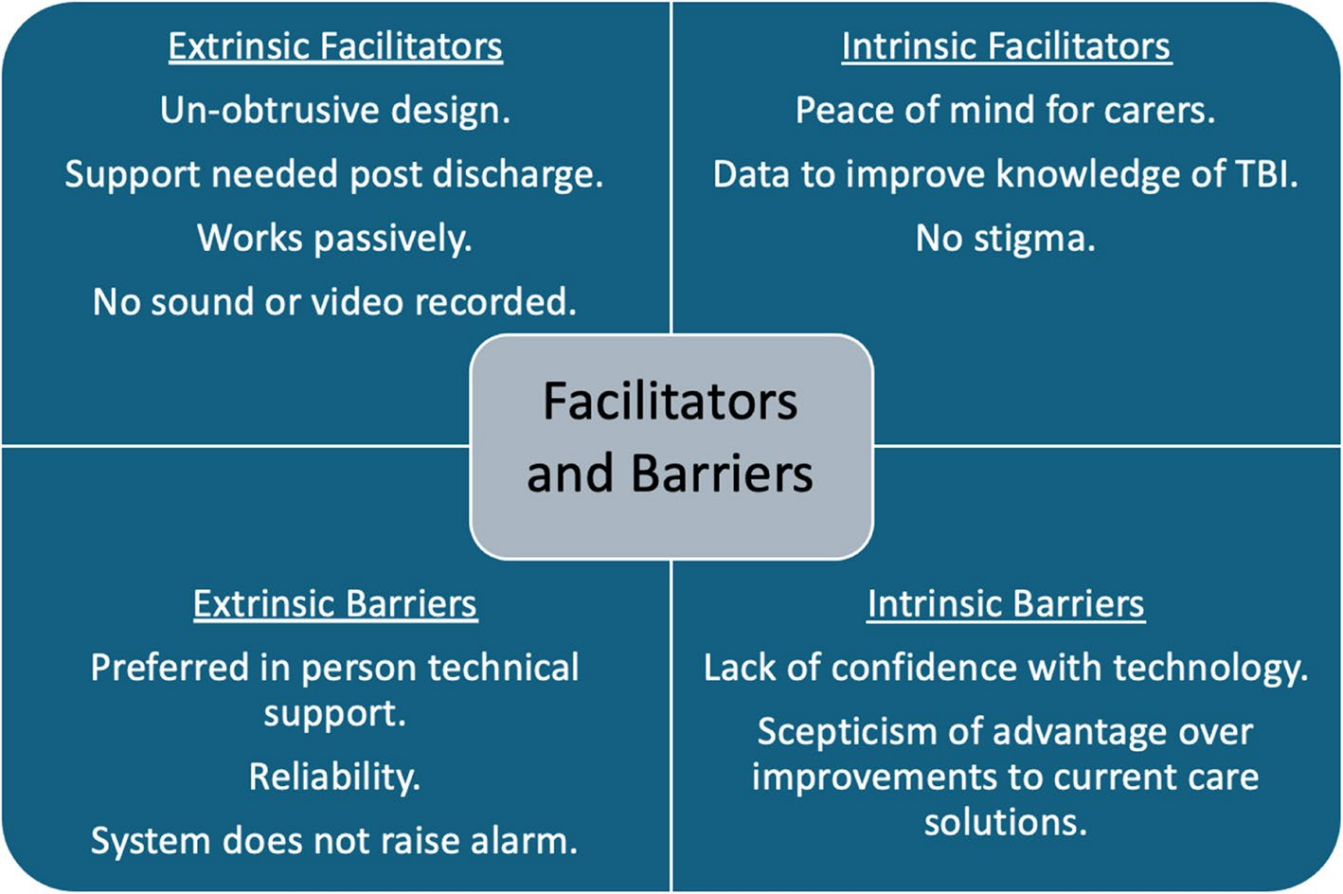
Facilitators and barriers to the use of home monitoring systems for older adults with TBI. Diagram demonstrating the key barriers and facilitators to the sustained adoption of home monitoring technology in older adult TBI that emerged from our analysis. Extrinsic factors refer to external influencing factors and intrinsic refers to person-specific factors

6 key themes were identified from the interviews (outlined in Fig. 3). A need to better understand TBI and support the transition from home to hospital drove participation in the study. Participants emphasised the importance of maintaining privacy and independence. Their experiences were influenced by their personal attitudes toward technology, as well as factors like ease of use and overall comfort with the system. The perceived need for and value of the system was a theme that came through for both patients and caregivers. Additionally, participants provided valuable suggestions for improving the system to better meet their needs. Each theme will be examined below with illustrative quotes, with additional illustrative quotes in Supplementary file 4. Factors affecting acceptability, barriers and facilitators are explored under each theme. The factors we found affecting the acceptability of home monitoring are outlined in Fig. 4. The main barriers and facilitators to adoption are summarised in Fig. 5.

### Theme 1: Improving understanding of TBI and support after discharge

Participants' concerns about post-discharge support and their limited understanding of TBI motivated them to take part in a home monitoring study. Most felt they had little or no knowledge of TBI:

*“What might happen in the future? Would it become worse? …. I thought Oh God, something else...... First diabetes and cancer and now this.”*

*(P1, male, age 86)*

This resulted in anxiety about the future and potential complications and the long-term effects of the TBI. This anxiety was compounded by limited support after discharge:

*“A lot of people are just left; mum has only had one follow up appointment. Since then, that’s it.”*

*(P5 study contact, female, age 54)*

It was also expressed by participants that TBI was a neglected area of research:

*“They do study people who have heart attacks but not brain injuries”*

*(P4 study contact, age 79)*

### Theme 2: Privacy and independence

#### Acceptability

Patients and study contacts did not perceive the home monitoring set up as an invasion of privacy. This included both physical privacy (the degree to which a person or their personal space is physically accessible) and informational privacy (sharing of personal information). They found passive infrared movement sensors acceptable, as they only recorded movement only, not video or sound:

*“Once I knew there were no cameras, I was fine. I just said to my daughter ‘I don’t want people watching me.”*

*(P3, female, age 67)*

Participants did not feel pressure to alter their behaviour e.g., increasing or limiting their activities in any way while being monitored:

*“I never thought, Oh dear! I’m not going to do that here because I’m being watched.”*

*(P1, male, age 86)*

Similarly, the presence of sensors did not affect daily routines with friends and family:

*“When you come in, it’s just like, you go into your mum’s house and then you leave. You don’t really look at the sensors or anything. Well, I don’t. I don’t know anybody else that does.”*

*(P5 carer, female, age 54)*

#### Barriers

Although most respondents had no privacy concerns some found the PIR sensors to be intrusive comparing them to “Big Brother”:

*“I think it’s a big burden. All this spying. I think when I pass them, ‘whatever are they doing there? Are they hatching a plot?”*

*(P4, male, age 79)*

However, this patient did not withdraw from the study as he felt the system may be “useful to help elders” and appreciated the reassurance their family had from the system.

#### Facilitators

Patients and study contacts appreciated the “peace of mind” offered by having the sensors in place:

*“Basically, giving my kids peace of mind. But I didn’t dislike them at all. Didn’t bother me.”*

*(P3, female, age 67)*

Additionally, perceived benefits of the system outweighed concerns over infringements of privacy:

*“I mean, I suppose some people might think that it could be used in a way that it was detrimental. I mean people could be spied on all the time, or something like that. I mean, I never thought of that, but maybe some people think that, but from my point of view, from the point of view of the person who's ill, I think it's very helpful”*

*(P8 study contact, female, 87)*

In terms of informational privacy, patients trusted the security measures and were happy for researchers and study contacts to access their sensor data: Study contacts within the household also all consented for their data to be used:

*“[data security], I don't care who knows what about me”*

*(P7 study contact, male, age 63)*

No social barriers like stigmatisation were reported, and the system did not negatively impact relationships with family, friends, or carers:

*“I don't mind what my family think of what I'm doing or anything really. I have a very good, close relationship with my son.”*

*(P9, male, age 87)*

Patients did not associate the monitoring equipment with poor health or with a loss of independence. Notably, patients who negatively associated walking aids and carers with ageing and ill health did not ascribe the same feelings towards the monitoring equipment. However, patients did not feel that the system directly improved their sense of independence.

### Theme 3: Older adults’ attitudes to technology and technology to monitor health

#### Acceptability

Participants' attitudes towards technology varied based on their experiences. Generally, patients and study contacts regularly used the internet, tablets, and smartphones. However, confidence and technical literacy levels varied impacting how acceptable participants found the technology to be.

#### Barriers

Some participants were less enthusiastic about fully engaging with technology:

*“I would say she’s afraid of it but she’s not afraid of it. She’s not bothered. On her phone, she only answers her calls or makes calls. If she gets a text message, she doesn’t read text messages. She waits for somebody to come to read the text messages. I mean she says at her age, she’s not really bothered about it. She’s not that fussed. Quite laid back really.”*

*(P5 study contact, female, age, 54)*

Others felt intimidated by the complexity of technology and felt no desire or need to engage with it:

*“I find that you don’t often understand the language [technology related]. I think a fairly significant portion of people our age is not techy. We have several friends who don’t send emails, who don’t have anything to do with the internet at all. They don’t understand it, and they don’t want to.”*

*(P1 study contact, female, age 86)*

Concerns about false notifications were also prevalent, with participants stressing the need for the system to report accurately to avoid unnecessary anxiety or misuse of emergency services:

*Patient: “If it’s showing on the screen that I stopped moving at a time when you wouldn’t expect me to stop or if I stopped moving to have a nap in the afternoon or something like that.”*

*Researcher: “and you would be concerned that it thinks something is wrong and an ambulance has been called?”*

*Patient: “yes”*

*(P9, male, age 87)*

Some study contacts experienced anxiety related to app usage, particularly about how to respond to potential problems and the accuracy of the data:

*“On the first day, when I woke up in the morning, there was a movement in the hallway, and then there was no other movement. But after that, I just relaxed because I realised that the sensors don’t always pick up all the movement, especially at night-time. But then, I would have to work out what is going on. It could be anything.”*

*(P1 study contact, female, age 86)*

#### Facilitators

Some participants felt confident using technology and valued the potential benefits such as health monitoring. For instance, some were using heath monitoring technology (Fitbit) before participating in the study:

*“I’m getting rather better at it. I can get it [fit bit] to monitor my sleep. They monitor everything but I only look at my sleep and the exercise I’ve done.”*

*(P1, male, age 86)*

Prior to entering the study, family members of pats with dementia also used technology informally to ensure safety, such as GPS trackers to monitor those at risk of wandering:

*“I’ve got one of these Air Tags which tells me where he is because I put it on his keychain.”*

*(P2 study contact, female, age 89)*

### Theme 4: Living with the monitoring equipment and ease of use

#### Acceptability

Respondents favoured the unobtrusive passive nature of the equipment, requiring minimal engagement. Wearable sensors were less tolerated. Respondents rejected them due to concerns about losing them or forgetting to charge them. Wearable users reported difficulties manipulating small buttons.

As it was a remote system, patients and study contacts were contacted via phone or email to troubleshoot technical issues. Participants varied in their confidence with troubleshooting, preferring a personalised approach based on their comfort level with technology. Some of the instructions given were seen to be technical and there were concerns about the intuitiveness of the user interfaces. When asked about their confidence troubleshooting, respondents reported that they would if instructions were pitched more appropriately:

*“As long as the language wasn’t too technical, and the instructions actually worked”.*

*(P1, male, age 86)*

#### Barriers

Some patients and study contacts preferred in-person site visits from the research team to manage technical issues:

*“I didn’t notice that that one [PIR motion sensor] wasn’t working…they sent it [PIR] through and then we changed it, and it didn’t work …she [MP] had to come in and do it. She had to go through to somebody to join it up into the network. So, it wasn't as straightforward as just pressing the button.”*

*(P5 study contact, female, age 54)*

Others found the app’s alert function difficult to turn off, leading to frustration:

*“They do know if you’re not moving around the house anyway. When I was going away for the week. My daughter had the sensor program on her phone, kept pinging that there was no movement. She said it would drive her off the wall.”*

*(P3, female, age 67)*

#### Facilitators

Some participants were comfortable troubleshooting themselves:

*“The sleeping mat [not working], it was moving, so I went and turned the mattress. But yeah, there wasn’t any maintenance.”*

*(P3, female, age 67)*

Patients and study contacts who were confident with technology chose to interact with the app.

For the study contacts who wanted to engage with the app, they found it easy to use:

*“Yes, very easy, it's got Howz on it. You just press it and then you get everything……No, there was no difficulty and when I couldn't get in it, I remembered that I put my email address in and then I put my password in.”*

*(P8 study contact, female, age 87)*

### Theme 5: The perceived value of home monitoring

#### Acceptability

Patients' views on the benefits of home monitoring varied based on their health, social setups, and care needs. For instance, one respondent who was functionally able and independent (Table 1) did not perceive any significant benefits from the current system:

*Patient: “It made no effect (to me) at all; I certainly did not dislike it”*

*Researcher: “Do you think you would recommend the system just on its own?”*

*Patient: “I think not.”*

*Researcher: “Can you expand on why?”*

*Patient: “Because I can’t see the benefit.”*

*(P1 male, age 86)*

Equally, those with strong support networks acknowledged less personal benefit but recognised its potential advantages for others:

*“I understand the research and what’s around it and the reason why I said to my mum. My mum said she wanted to do it, and I said yeah “I agree” is because she’s got a good support system. and there’s a lot of people that don’t have that, so you must have something to compare so for me, we still spend a lot of time here. A lot of time there’s somebody else here with them. She’s spending more time now without assistance but… The effectiveness, I suppose it would be good. If she did have it permanently, in the house, for example, and she fell, and it was just her and my dad. Probably I would be looking at it and monitoring as well also. I do think that it could be effective.”*

*(P5 study contact, female, age 54)*

All Interviewees wanted the system to address their own specific needs. Getting help in the event of a fall was a key concern for all respondents. For the technology to gain wider acceptance, participants felt it should reliably detect emergencies, and then facilitate communication with health and social care providers.

#### Barriers

A significant barrier identified was the lack of an integrated alarm-raising feature:

*“I think the answer is no that I wouldn’t recommend it. I certainly would if there was a way of dialling into it.... Of raising an alarm.”*

*(P1, male, age 86)*

Study contacts actively involved in caregiving saw less daily benefit from the system, doubting its efficacy compared to their existing care setup:

*“I'm with her, I'm holding on to her, you know, so given that I feel like I kind of can account for almost every moment of her life, you know, by being there or knowing exactly where she is and what she’s doing. I'll be interested to see if the data shows me something, you know, that I'm not aware of.”*

*(P7 study contact, male, age 63)*

#### Facilitators

Respondents identified several potential uses for the system: providing reassurance to caregivers, particularly for those who live alone or are more vulnerable; monitoring progress post-hospital discharge; and offering valuable insights for researchers and clinicians studying the effects of TBI in older adults. For patients living alone or far from family, the system offered significant reassurance through remote monitoring by family members and researchers:

*“Like going out in the middle of the night and things like that. The example is when my daughter phoned me. Said [daughter] the sensor [door sensor] had gone off early in the morning. I was doing unusual things. You don’t know what’s going to happen and you don’t know your outcome.”*

*(P3, female, age 67)*

Study contacts' perceptions were influenced by their circumstances; those with a greater need for home monitoring responded more positively. Study contacts of patients living alone, with cognitive impairment, and higher care needs found the system particularly valuable:

*“Because of having them, I realised that one day he was out for six hours, so something must have happened, and we were able to find out that he had another sort of little seizure. I found it very helpful. I knew what was happening to him. Because I live far away, because he relies on me a lot, it was extremely helpful. I could tell when he got up, when he went out, when he had a walk, when he came back, when he put the kettle on in the evening, or when he woke up during the night. I thought that was very useful.”*

*(P8 study contact, female, age 87)*

### Theme 6: Improvements to the system

Interviewees felt there could be enhancements to improve personal safety, particularly to address fall risks. They recommended a system that detects balance changes to help prevent falls and help if one occurs:

*“A system that tells one about one’s steadiness from the point of view of the research, it would be interesting to know if people’s strides were getting better or if they became unsteady.”*

*(P1, male, age 86)*

Other preventative measures included a voice alarm at the top of the stairs to alert those with cognitive impairment to the potential dangers:

*“One of the best things we did was having that funny thing on the wall that said “It’s the middle of the night. Don’t go out.” When he [P4] came back from the hospital, the first night, he went all the way to the top of the stairs with no lights on or anything.”*

*(P4 study contact, female, age 79)*

Participants also suggested they would value a user-friendly way to communicate through the system to be able to ask for help when it was needed. Ways of raising an alarm included pressure sensors in the flooring to alert of a fall. Proposed alarm-raising mechanisms included pressure sensors in the flooring to alert of a fall. An additional suggestion from a family member was incorporating cameras that activate in response to abnormal movements detected by sensors:

*“If you can combine that with the camera, you know, so you get the sensor alert, then you check on the camera and you got the complete sort of everything you need.”*

*(P7 study contact, male 63)*

Interviewees also felt the system could improve home safety by using sensors on implements like the cooker:

*“You monitor the use of microwaves, cookers and things like that? That's the only thing. Anything that can cause danger. Any way of monitoring that sort of thing, if they had to cook a meal or prepare. Mum cooks.”*

*(P5 carer, female, age 54)*

Additionally, technology to improve medicine safety by including a sensor on a medication locker was suggested for those with cognitive impairment:

*“Something that could trigger if you haven't given the medicine today would be, I think, a big deal.”*

*(P7 study contact, male, age 63)*

## Discussion

We explored the experiences of patient and their families and caregivers with home monitoring after hospitalisation with TBI, focussing on understanding acceptability, facilitators and barriers to use. Most participants found the equipment acceptable because they recognised the care gap and need for it and appreciated its minimal engagement requirements. The technology was particularly valued by caregivers of older adults living alone or with frailty and cognitive impairment. Our results highlight there are challenges and barriers to be addressed in future home monitoring research, including unfamiliarity with technology, scepticism of benefits over existing care solutions, and concerns about its reliability in detecting emergencies like falls. However, we identified key factors that would encourage widespread use of passive home monitoring such as the reassurance it provided to caregivers, its minimal impact on privacy and the technology’s potential to provide “a road map” for caring for older adults with TBI.

### Acceptability of home monitoring technology

Older adults and caregivers had broadly positive experiences with home monitoring equipment after hospital discharge following acute traumatic brain injury (TBI). The equipment was well tolerated by both groups, including caregivers living with patients. Patients and caregivers found it acceptable for several reasons: they saw a need for it, it could benefit them, and its passive unobtrusive design made it easy to use in their homes.

Our study further highlights home monitoring is acceptable as a means to bridge unmet care needs [30, 45]. For instance, caregivers had often already adopted health monitoring tools (e.g., Apple Air Tags, "nanny cams," pendant alarms), reflecting current pressures on home care services and the increasing role of family caregivers [59]. The need for greater support after discharge following TBI was a key motivator for engaging with home monitoring technology.

We found that individual differences, such as level of frailty, functional status and cognitive function, are likely to impact acceptability. Passive monitoring systems are particularly attractive for vulnerable older adults and caregivers, where poor cognition may affect insight and recall [1, 60]. However, the system was less acceptable to those with good support systems and good health. This suggests a need for appropriately prescribed and customisable technology. A “one size fits all” approach is unlikely to work, particularly for the heterogeneous older adult TBI population [8].

Most participants found the passive home monitoring system acceptable as it was unobtrusive, preferring systems requiring minimal user input, consistent with existing literature [25–27, 31]. Only three patients used the wearable activity watch, all finding it difficult to use, supporting literature that complex devices risk abandonment [30, 56].

### Barriers to adopting home monitoring technology

Potential barriers included the reliability of the technology, lack of in-built alerts system, a preference for in-person technical support, poor confidence with technology and scepticism of the advantage over current care solutions.

With regards to the reliability of the technology, false notifications on study contacts apps raised concerns, highlighting the need for consistent device performance to ensure user trust and continued use, as highlighted in previous studies [8, 55]. In addition, the inability to raise an alarm using the system may deter its use, emphasising that older adults prioritise technology that is capable of detecting emergencies in real time, such as falls [43]. This is particularly relevant to older adult TBI, where falls and multiple TBI in older age groups carries a poor prognosis [23].

Most participants preferred in-person support for technical issues, which could be a barrier if not feasible, especially for those with limited support networks. Our findings further identified the need to consider who is best placed to provide support in maintaining these systems as they undergo further development [57]. Simplified instructions could improve comfort in managing technical problems, highlighting that more Patient and public involvement work is necessary to ensure accessibility for older adult users [8, 30, 44].

Contrary to some studies, our findings suggest a positive shift in older adults' attitudes toward technology, showing engagement [28, 58, 61–65]. However, while participants engaged with technology, comfort levels varied. Poor technical literacy remains a barrier and future work should focus on education and user-friendly designs [66, 67].

Additionally, perceived futility of the home monitoring system, in particular lack of any advantage over current care solutions, were barriers to use. Some participants did not see how the system would directly benefit them or improve their existing care arrangements, which aligns with findings from other studies [60, 68, 69]. Equally, the system neither promoted nor detracted from participants' sense of independence contrary to other study findings [44, 61, 70]. However, this could reflect the system's developmental stage and lack of real-time monitoring.

### Facilitators to adopting home monitoring technology

Key factors that would facilitate the adoption of home monitoring technology included a need for improved understanding and support following traumatic brain injury (TBI). Participants appreciated the reassurance it provided to caregivers and had minimal concerns about privacy or stigma.

In line with previous studies, participants lacked knowledge about TBI and were apprehensive about what to expect post-injury [71, 72]. The opportunity to contribute to a better understanding of TBI was a strong motivator for using home monitoring during recovery. Our findings underscore the need for more comprehensive support and information for older adults and their caregivers after TBI and highlights the importance of investing in research for older adult TBI [23, 73, 74]. There was notable perceived need for additional support during the vulnerable period after hospital discharge following TBI. Therefore, remote home monitoring could be a valuable tool help bridge a care gap in health systems with limited resources for extended follow up.

Privacy concerns, often noted as a barrier [29, 60, 63, 66], were not significant in our cohort likely due to the unobtrusive passive nature and lack of visual recording. Our findings add to existing literature that participants had fewer concerns about privacy in the absence of video or sound recording [27, 31, 57, 63, 75]. Patients and cohabiting caregivers were comfortable sharing their data with researchers and healthcare professionals. While previous studies indicated that older adults might worry about losing control over their daily activities or causing undue concern for caregivers [63], our cohort did not express these concerns. Participants were willing to share data when they understood its purpose, aligning with findings that privacy concerns are not a significant barrier to adopting home monitoring technology [63, 64, 75].

Additionally, participants did not perceive stigma associated with using the equipment due to its unobtrusive design and minimal user input required. This aspect facilitated the acceptance of passive home monitoring This contradicts some studies that suggest older adults adopt technology begrudgingly or as a last resort [43, 61, 63].

### Limitations

The version of Minder used in our study is still under development and was not live monitored by the research team, which influenced respondents’ opinions on its utility. However, even when the perceived utility was low, the system was considered tolerable.

Most participants were White, with English as their first language. The study sample, though diverse in age, socioeconomic status, care requirements, and social environment, was small and recruited from a single specialist centre, limiting generalisability. Also, responses reflect the acute phase of illness and may not translate to chronic condition monitoring.

Researcher involvement in planning and delivery may introduce confirmation bias. None of the researchers, however, had prior experience using home monitoring technology or similar equipment in their clinical practice, which could have influenced data collection and analysis. For practical reasons, interviews were conducted by researchers already known to the participants, which may have introduced bias. Also, volunteer participants may already be positively predisposed to the technology. Nonetheless, participants expressed both positive and negative views of the system. Interviews with study contacts and participants were sometimes conducted together, which may have led to contamination of opinions. Conversely, these discussions or disagreements may have enhanced the richness of our dataset.

### Future directions

Based on our findings, development of future systems should emphasise safety features, like fall detection and prevention, and be customised to older adults’ individual needs. Studies using home monitoring technology could leverage the potential of machine learning to create personalised profiles that can identify changes in health based on individual trends [6]. Equally, future work should seek to optimise system reliability and minimise false alarms, whilst exploring the feasibility of real-time monitoring with phone call feedback and alerts.

As our participants disliked remote technical support, conducting a health economic analysis of the equipment within current healthcare systems, including the cost of in-person technical support, would be valuable. Additionally, research should examine older adults' understanding of data security; while there is a willingness to share data, this may indicate limited awareness of cyber security issues [76]. Finally, future qualitative work should gather insights from community care providers, who are key stakeholders in this technology.

## Conclusion

This study demonstrates a broadly positive reception of home monitoring technology among older adults and caregivers following hospital discharge after acute traumatic brain injury (TBI). There was a willingness to embrace new technology to provide peace of mind for caregivers and improve knowledge of TBI. Participants favored the contactless passive system and absence of cameras. Home monitoring may be particularly beneficial for individuals who live alone or have cognitive impairments. Addressing barriers to implementation and tailoring systems to older adults’ individual needs will be essential for broader adoption and effectiveness of passive home monitoring. For sustained use, the technology should be developed in conjunction with older adults and caregivers and be developed with older adults’ specific needs in mind.

## Supplementary Information

Supplementary Material 1.

## Data Availability

The datasets utilized and/or examined in the present study can be obtained from the corresponding author upon request. Email mparkin1@ic.ac.uk.
