# Supplementary material for: Experiences with home monitoring technology in older adults with traumatic brain injury: a qualitative study
Source: BMC Geriatr. 2024 Sep 30;24:796. doi: 10.1186/s12877-024-05397-0 (PMC11440809; doi:10.1186/s12877-024-05397-0)
Supplement: Supplementary file 1 — Supplementary Material 1. [file 12877_2024_5397_MOESM1_ESM.docx]

**Supplementary materials**

**Supplementary file 1:** Participants and study contacts interview guides

**Supplementary file 2:** Framework for thematic analysis

**Supplementary file 3:** COREQ checklist

**Supplementary file 4:** Summary of themes with additional illustrative participant quotations

**Supplementary file 5:** Acknowledgement list for UK Dementia Research Institute (UK DRI) Care Research & Technology (CR&T) Centre publications using the MINDER core data set

**Supplementary file 1**

**Participants Topic guide.**

- **Introduction**
- Thank you for agreeing to participate in this interview. The aim of this interview is to understand your experience participating in the Monitoring after TBI (maTBI) study. Your answers will be available to the rest of the study team, who will maintain confidentiality. Your answers will be anonymous when published. The interview will be recorded, and I may take some notes whilst you are talking. You do not have to answer any questions you do not feel comfortable with, and we can stop the interview at any time. Requesting to stop the interview or declining to answer questions will not have any impact on your medical care.

- **Background**
- Before your injury, what did you already know about traumatic brain injuries?
- What, if any, aspects of a traumatic brain injury where you worried about?
- Before this study, how familiar were you with the type of technology used in this study (motion detectors, sleep mat, and wearable)?
- At the start of this study, how comfortable/confident did you feel about using the technology (sleep mat/wearable)?

- **Acceptability/Social Amiability**
- Did anyone comment on the sensors/bed/or watch?
- What did you think of what they said?
- Was it surprising to you?
- How did you feel about having visitors in your home after the technology was installed?
- *(if answer indicated hesitancy in having visitors)* How did that affect or alter your behavior around having visitors to your home?
- Were these feelings primarily related to the technology or to the injury itself?
- Did you have any concerns about interacting with the wearable in front of others?

- **Personal Impact**
- What has been the impact of this sensor system on your daily life?
- Did you generally like or dislike having this sensor system?
- What aspects of having the sensor system did you like/dislike?
- Do you generally feel positively or negatively towards the sensor system?
- *If not already answered:* Did monitoring your condition cause you to be stressed or anxious? Secure? Indifferent? No Impact?
- How has this system impacted your sense of independence after your injury, if at all?
- How has this system affected the way you see yourself after your injury, if at all?

- **Ease of Use / Troubleshooting abilities**
- What was your experience using the watch?
- Which aspects did you like?
- Which aspects did you dislike?
- Were there any aspects that made it difficult to use?
- What was your experience having the infrared monitors?
- Which aspects did you like?
- Which aspects did you dislike?
- What was your experience having the bed mat?
- Which aspects did you like?
- Which aspects did you dislike?
- Did you encounter any technological difficulties?
- What was the issue(s)?
- How often did you encounter the issue(s)? (e.g. daily, weekly. rarely, once, etc.)
- What was your experience troubleshooting the problem(s)?
- What was your experience with maintenance for the system (as a whole)?
- Was it practical in terms of time needed?
- How easy or difficult did you find it?

- **Perceived Effectiveness**
- What are your thoughts on the sensors’ effectiveness in detecting falls/unexpected events for people like you, who have had a brain injury?
- How do you feel the system can be improved to better detect falls/unexpected events for TBI patients?
- If you were to rate the 3 measures (bed mat, motion detectors, wearable) from most to least effective at picking up health problems, how would you rate them? Why?

- **Suggestions for Improvement: specific to the system**
- What was your experience with making it to your in-person monitoring appointments?
- Did you miss any? If so, why? If not, were there any significant challenges that you experienced regularly getting here?

**OR What** was your experience of having us conduct home visits?

- Would you have preferred lab visits?
- Did you have an overall positive or negative experience with the weekly phone calls?
- How much did the weekly phone calls contribute to your overall experience of the system?
- If this system gets widely rolled out, it would only be the home monitoring parts (watch, bed mat, motion sensors). There wouldn’t be a 3-week visit or weekly phone calls. Would you recommend such a system to other people who had just left hospital with a TBI?
- Why/why not?
- What would you tell them about the system?
- Currently, here are the things we hope to eventually pick up with this system: activity patterns as they relate to falls, unusual events (such as agitated behavior, etc.), and prognosis (how well the patient recovers based on their injury). Is there something that you wish the system could detect that it does not in its current form?

- **Experience participating in the Study: specific to our team/the research process**
- What motivated you to take part in the study?
- What aspects of the study put you off taking part?
- How were those dealt with, if at all, prior to you taking part?
- What benefits, if any, did you expect to experience by taking part in the study?
- What challenges, if any, did you anticipate by taking part in the study?
- Did you at any point in the study consider discontinuing?
- Why so?
- What made you decide to continue?
- How did you find the process of being asked to take part in the trial?
- How did you feel about the amount of information you received in the study? Was it too much/too little; too frontloaded/to spread out?
- How did you feel about the way in which the team communicated the information to you?
- How well did you understand the trial from the information you received?
- How did you find the support you received with the devices?

- **Miscellaneous**
- Is there anything else that you would like to share?

**Study contacts topic guide**

- **Introduction**
- Thank you for agreeing to participate in this interview. The aim of this interview is to understand your experience as a loved one of a participant in the Monitoring after TBI (maTBI) study. Your answers will be available to the rest of the study team, who will maintain confidentiality. Your answers will be anonymous when published. The interview will be recorded and I may take some notes whilst you are talking. You do not have to answer any questions you do not feel comfortable with, and we can stop the interview at any time. Requesting to stop the interview or declining to answer questions will not have any impact on your loved one’s medical care.

- **Background**
- Before your loved one’s injury, what did you already know about traumatic brain injuries?
- What, if any, aspects of a traumatic brain injury where you worried about?

- **Acceptability/Social Amiability**
- Did anyone comment on the sensors/bed/or watch?
- What did you think of what they said?
- Was it surprising to you?
- How did you feel about having visitors in your home after the technology was installed?
- *(if answer indicated hesitancy in having visitors)* Were these feelings primarily related to the technology or to the injury itself?
- What impact has this system had on your loved one’s ability to interact with others?
- Prior to this injury, did your loved one have any stigmas surrounding aid devices?

- **Personal Impact**
- What has been the impact of this sensor system on your loved one’s daily life?
- From your point of view, how did your loved one generally feel about the sensor system?
- *If not already answered:* Did monitoring the condition cause your loved one stress or anxiety? Secure? Indifferent?
- How do you think this system has impacted your loved one’s sense of independence after the injury, if at all?
- How do you think this system has affected the way your loved one sees him/herself after the injury, if at all?

- **Ease of Use / Familial Impact**
- What has been the impact of this sensor system on your daily life?
- What aspect(s) of the system presented the most difficulty for you/the family?
- How much time did the system require of you?
- How did you feel about being included in the monitoring?
- If not already implied: Did it make you uncomfortable at all?
- How did the system impact your relationship with your loved one, if at all?

- **Perceived Effectiveness**
- What are your thoughts on the sensors’ effectiveness in detecting falls/unexpected events for people like your loved one who have had a brain injury?
- How do you feel the system can be improved to better detect falls/unexpected events for people like your loved one who have had a brain injury?
- If you were to rate the 3 measures (bed mat, motion detectors, watch) from most to least effective at detecting events, how would you rate them? Why?

- **Suggestions for Improvement: specific to the system**
- Did your loved one have an overall positive or negative experience with the weekly phone calls?
- How much did the weekly phone calls contribute to your loved one’s overall experience of the system?
- If this system gets widely rolled out, it would only be the home monitoring parts (watch, bed mat, motion sensors). There wouldn’t be a 3-week visit or weekly phone calls. Would you recommend such a system to other people who had just left hospital with a TBI?
- Why/why not?
- What would you tell them about the system?
- Currently, here are the things we hope to eventually pick up with this system: activity patterns as they relate to falls, unusual events (such as agitated behavior, etc.), and prognosis (how well the patient recovers based on their injury). Is there something that you wish the system could detect that it does not in its current form?

- **Experience participating in the Study: specific to our team/the research process**

*Questions about participating in the study:*

- What did you think of your loved one’s decision to take part in the study?
- Did you have any concerns about your loved one participating in the study?
- Were there any aspects of the study that your loved one complained about/was bothered by?
- What aspects of the study put your loved one off taking part?
- How were those dealt with, if at all, prior to him/her taking part?
- Did your loved one at any point in the study consider discontinuing?
- Why so?
- What made him/her decide to continue?

*Questions about our team:*

- How did you find the process of being asked to take part in the trial?
- How did you feel about the amount of information you received in the study? Was it too much/too little; too frontloaded/too spread out?
- How did you feel about the way in which the team communicated the information to you?
- How well did you understand the trial from the information you received?
- How did you find the support you received with the devices?

- **Miscellaneous**
- Is there anything else that you would like to share?

**Supplementary file 2**

**Framework for thematic analysis**

**1 Participant and carers experience of TBI**

1.1 Perceptions of TBI

1.2 Communication with Clinical teams about TBI

1.3 Experience of inpatient care

1.4 Impact of injury on function

1.5 Perceptions of outpatient clinical care and follow up

**2 Perceptions of using technology to monitor health**

2.1 General attitudes towards technology

2.1 Prior knowledge of home monitoring technology

**3 Acceptability/Social Amiability of the home monitoring system**

3.1 Privacy

3.2 Feelings of self-consciousness about sensors

- 1. Awareness of the sensors

3.4 Visitors’ perception of the sensors

3.5 Impact of system on family care and relationships

**4 Experiences using the home monitoring system**

4.1 Family/carer experiences of the system

4.2 Participants experiences of the system

4.3 Ease of use

4.4 Experience with troubleshooting & support

4.5 Robustness of sensors in home environment

4.6 Social factors that influence potential clinical efficacy of system

4.7 Patient changing feelings about necessity/role of system as a whole

4.8 Perception of benefits of the system

4.9 Relative benefit of system for family versus patient

4.10 Perception of system efficacy in detecting ill health or clinical events i.e. falls

4.11 Particular aspects of the monitoring system that could be most helpful

**5 Monitoring system uses and potential improvements**

5.1 How systems can be improved to meet specific care needs

5.2 Thoughts on the potential uses of the system

**Supplementary file 3**

**COREQ (COnsolidated criteria for REporting Qualitative research) Checklist**

A checklist of items that should be included in reports of qualitative research. You must report the page number in your manuscript where you consider each of the items listed in this checklist. If you have not included this information, either revise your manuscript accordingly before submitting or note N/A.

| **Topic** | **Item No.** | **Guide Questions/Description** | **Reported on Page No.** |
| --- | --- | --- | --- |
| **Domain 1: Research team and reflexivity** |  |  |  |
| *Personal characteristics* |  |  |  |
| Interviewer/facilitator | 1 | Which author/s conducted the interview or focus group? | 4 |
| Credentials | 2 | What were the researcher’s credentials? E.g. PhD, MD | 4 |
| Occupation | 3 | What was their occupation at the time of the study? | 4 |
| Gender | 4 | Was the researcher male or female? | 4 |
| Experience and training | 5 | What experience or training did the researcher have? | 4 |
| *Relationship with participants* |  |  |  |
| Relationship established | 6 | Was a relationship established prior to study commencement? | 8,21 |
| Participant knowledge of the interviewer | 7 | What did the participants know about the researcher? e.g. personal goals, reasons for doing the research | 8 |
| Interviewer characteristics | 8 | What characteristics were reported about the inter viewer/facilitator? e.g. Bias, assumptions, reasons and interests in the research topic | 21 |
| **Domain 2: Study design** |  |  |  |
| *Theoretical framework* |  |  |  |
| Methodological orientation and Theory | 9 | What methodological orientation was stated to underpin the study? e.g.  grounded theory, discourse analysis, ethnography, phenomenology, content analysis | 4 |
| *Participant selection* |  |  |  |
| Sampling | 10 | How were participants selected? e.g. purposive, convenience, consecutive, snowball | 5 |
| Method of approach | 11 | How were participants approached? e.g. face-to-face, telephone, mail, email | 5 |
| Sample size | 12 | How many participants were in the study? | 5 |
| Non-participation | 13 | How many people refused to participate or dropped out? Reasons? | 5 |
| *Setting* |  |  |  |
| Setting of data collection | 14 | Where was the data collected? e.g. home, clinic, workplace | 5 |
| Presence of nonparticipants | 15 | Was anyone else present besides the participants and researchers? | 5 |
| Description of sample | 16 | What are the important characteristics of the sample? e.g. demographic data, date | 8 |
| *Data collection* |  |  |  |
| Interview guide | 17 | Were questions, prompts, guides provided by the authors? Was it pilot tested? | 5,6 |
| Repeat interviews | 18 | Were repeat inter views carried out? If yes, how many? | 6 |
| Audio/visual recording | 19 | Did the research use audio or visual recording to collect the data? | 6 |
| Field notes | 20 | Were field notes made during and/or after the inter view or focus group? |  |
| Duration | 21 | What was the duration of the inter views or focus group? | 8 |
| Data saturation | 22 | Was data saturation discussed? | 6 |
| Transcripts returned | 23 | Were transcripts returned to participants for comment and/or | 6 |
| **Topic** | **Item No.** | **Guide Questions/Description** | **Reported on Page No.** |
|  |  | correction? |  |
| **Domain 3: analysis and findings** |  |  |  |
| *Data analysis* |  |  |  |
| Number of data coders | 24 | How many data coders coded the data? | 6 |
| Description of the coding tree | 25 | Did authors provide a description of the coding tree? | 6 |
| Derivation of themes | 26 | Were themes identified in advance or derived from the data? | 6,8,9 |
| Software | 27 | What software, if applicable, was used to manage the data? | 6 |
| Participant checking | 28 | Did participants provide feedback on the findings? | 6 |
| *Reporting* |  |  |  |
| Quotations presented | 29 | Were participant quotations presented to illustrate the themes/findings?  Was each quotation identified? e.g. participant number | 8-18 |
| Data and findings consistent | 30 | Was there consistency between the data presented and the findings? | 8-18 |
| Clarity of major themes | 31 | Were major themes clearly presented in the findings? | 8-18 |
| Clarity of minor themes | 32 | Is there a description of diverse cases or discussion of minor themes? | 8-18 |

Developed from: Tong A, Sainsbury P, Craig J. Consolidated criteria for reporting qualitative research (COREQ): a 32-item checklist for interviews and focus groups. *International Journal for Quality in Health Care*. 2007. Volume 19, Number 6: pp. 349 – 357

**Once you have completed this checklist, please save a copy and upload it as part of your submission. DO NOT** **include this checklist as part of the main manuscript document. It must be uploaded as a separate file.**

**Supplementary file 4**

| **Theme** | **Illustrative quotation** |
| --- | --- |
| Improving understanding of TBI and support after discharge  Facilitators: | *“I was worried about how life would be after this.”*  *(P4 carer, female, age 79)*  *“I didn’t come around until much later, but I was concerned. My previous high velocity life was going to be altered.”*  *(P4, male, age 79)*  *“For example, my mother had a stroke, and I knew she had problems trying to get herself moving again...... I had heard of these things.”*  *(P6, female, age 63)*  *“The one thing I hadn’t appreciated before that they [hospital team] made a point to me, that repeated injuries would have a cumulative effect, not just an individual instance that you have to worry about.”*  *(P7 study contact, male, age 63)*  *“Participants: The [home monitoring] trial in the way helping with the problem I originally had [TBI]the help that I got from the specialist who were dealing with me in the hospital was pretty helpless. They didn’t say who they were or what they were and never listened to what I said.*  *Study Contact: One thing we did find was we never heard from the surgeon [neurosurgeon]. We talked to him for a long time, then never spoke to him again.”* |
| Privacy and independence  Facilitators:  Barriers: | *“I just went to bed when I wanted to go to bed.”*  *(P3, female, age 67)*  *“When I was first asked about it, I thought it would be cameras and that's why I said to my daughter “I don’t want cameras” and she said, “No it’s not cameras.” Once I knew there were no cameras yeah, I was fine. I just said to my daughter “I don’t want people watching me” and she said, “No they’re not mum.”*  *(P3, female, age 67)*  *“We’ve told people, if people are in here that we’ve got these sensors”*  *(P6, female, age 63)*  *Nobody noticed, literally, I would have to say you know that thing we're doing, that thing, what thing?*  *(P7 study contact, male, age 63)*  *There’s a huge line on the kitchen wall. Which I don’t quite understand, it’s quite intrusive. There are quite a few of them.*  *(P4, male, age 79)* |
| Older adults’ attitudes to technology and technology to monitor health  Facilitators: | “There’s *a[baby] monitor there, there's another one in there, everybody in the family, can connect to those monitors”*  *(P7 study contact, male,63)* |
| Living with the monitoring equipment and ease of use  Acceptability:  Barriers: | *“You know, it wasn't a problem. If there had been (a problem), It wouldn't have been a problem for me to point it out.”*  *(P9, male, age 87)*  *“Yeah, a couple fell off the wall and things like that. Oh, well but what I thought would be a minor inconvenience to the system, really. But, you know, I didn’t worry too much if it fell off and it wasn’t difficult to stick it back on again anyway. I just placed it where it could do the job.  They stand on their sides quite happily. “*  *(P9, male, age 87)*  *“The only thing I thought was, this idea of sticking them on the wall might not be so good because when you try to unstick them, you can pull the paint off the wall.”*  *(P9, male, age 87)* |
| The perceived value of home monitoring  Barriers: | *“It would be very useful to use, as you say, for detecting falls, but I don’t see how it would do it as such. Unless there was only one person in the house, and there was a big change in the movement pattern. I don’t see how you could improve it.”*  *(P1, male, age 86)*  *“Even if they knew something was wrong or anything there’s nothing, they could do about they’re miles away, or if not miles away, ones in reading, but she's disabled and she never comes to London.”*  *(P9, male, age 87)*  *“It meant that, I could tell everything was normal. I know their daily rhythms and I could tell that something is not right”.*  *(P5 study contact, age 54)* |
| Improvements to the system | *“Well, I suppose, similar to the way you have the sensor under the mattress, I don't know whether, similar sensors could be used elsewhere, under rugs or I don't know I'm a bit difficult to imagine it how it might work you know if someone's in a wrong place and falls over is the sensor in the right place to pick it up, what kind of flooring of you got, is it going to vibrate or not as it concrete?  but I mean but something like that would say a really heavy fall happens.”*  *(P7 study contact, male 63)* |

**Supplementary file 5**


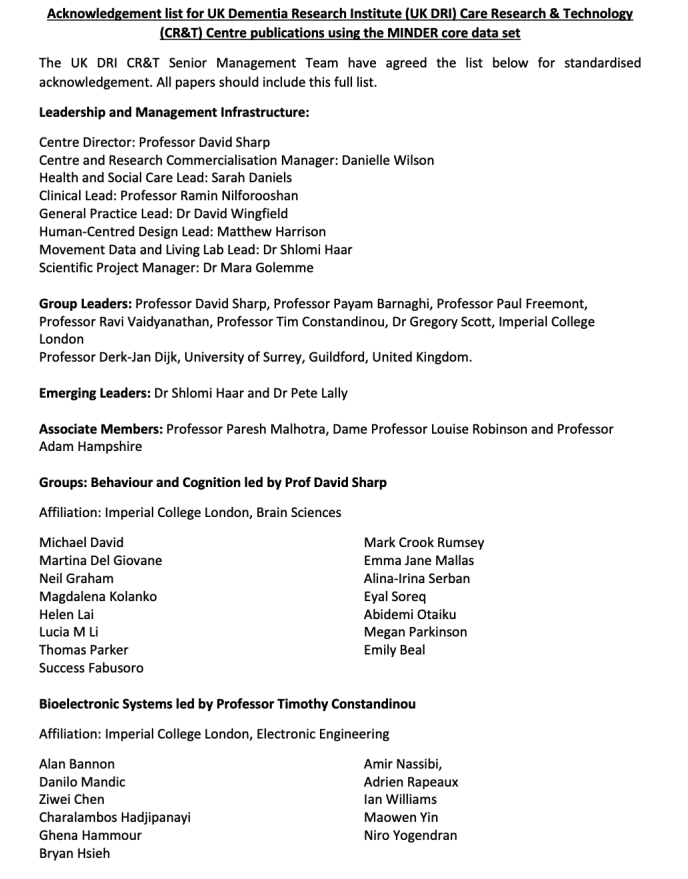


**
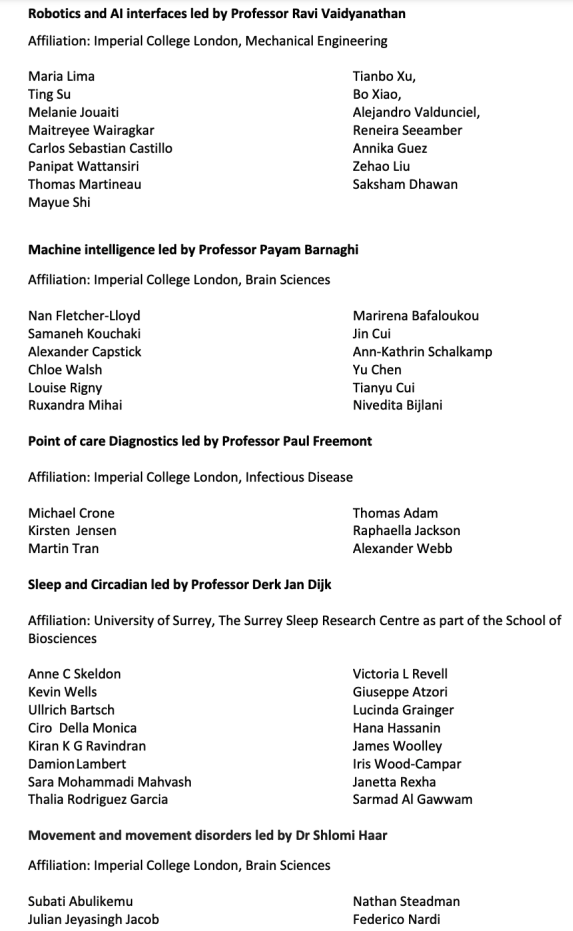
**

**
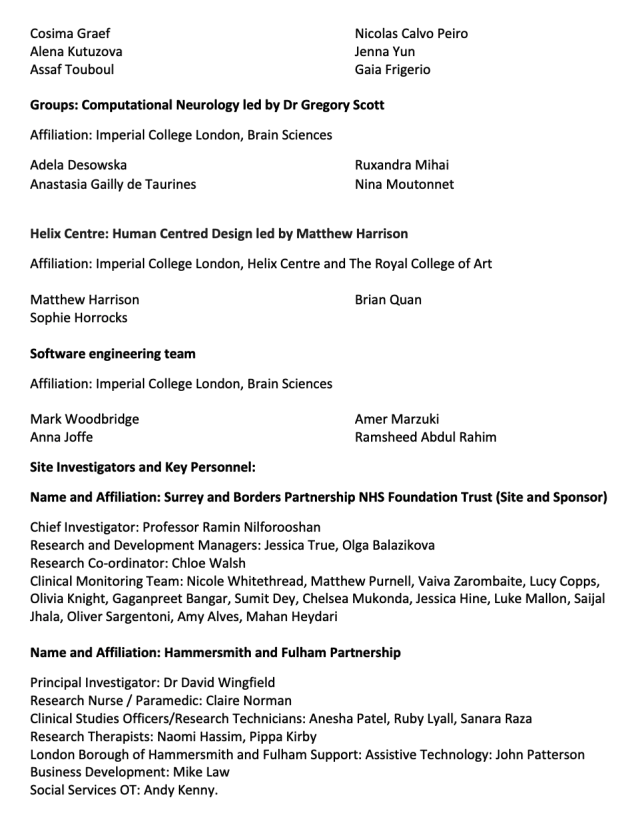
**
